# Can the SCD test and terminal uridine nick-end labeling by flow cytometry technique (TUNEL/FCM) be used interchangeably to measure sperm DNA damage in routine laboratory practice?

**DOI:** 10.1186/s12610-019-0098-2

**Published:** 2019-12-26

**Authors:** Cécile Grèze, Aline Guttmann, Hanae Pons-Rejraji, Marie-Paule Vasson, Jacqueline Lornage, Lemlih Ouchchane, Florence Brugnon

**Affiliations:** 1 Assistance Médicale à la Procréation, CECOS, Pôle Femme et Enfant, CHU Estaing, Clermont-Ferrand, France; 2 Hôpitaux Universitaires de Strasbourg, Laboratoires de Biologie de la Reproduction et de Diagnostic préimplantatoire, Strasbourg, France; 3 Biostatistics Unit, Department of Public Health, Clermont-Ferrand University Hospital, Clermont-Ferrand, France; 4 Institut Pascal, UMR 6602–CNRS/UCA/SIGMA, Image Guided Therapy/PEPRADE, Université Clermont Auvergne, Clermont-Ferrand, France; 5 Imagerie Moléculaire et Stratégies Théranostiques, U 1240 Inserm, Université Clermont Auvergne, Clermont Ferrand, France; 6 Unité de Nutrition Humaine, UMR 1019 – INRA/UCA, Equipe ECREIN, Université Clermont Auvergne, Clermont-Ferrand, France; 7 Unité de Nutrition, Centre de Lutte contre le Cancer Jean-Perrin, Clermont-Ferrand, France; 8 Service de Médecine de la Reproduction, Hôpital Femme-Mère-Enfant, CHU de Lyon, Bron, France

**Keywords:** Andrology laboratory, DNA damage, Flow cytometry, Spermatozoa, Sperm chromatin dispersion test, TUNEL, Laboratoire d’andrologie, altération de l’ADN spermatique, cytométrie en flux, spermatozoïdes, test de dispersion de la chromatine spermatique, TUNEL

## Abstract

**Background:**

Numerous tests have been proposed to evaluate sperm DNA integrity. To assess the sperm chromatin dispersion (SCD) test in an andrology laboratory, twenty-five men attending Clermont-Ferrand (France) University Hospital’s Center for Reproductive Medicine were recruited. Sperm DNA damage was measured in the same semen samples using the SCD test and the Terminal Uridine Nick-end Labeling by flow cytometry technique (TUNEL/FCM) after density gradient centrifugation.

**Results:**

SCD test reliability between readings, readers or slides was clearly established with very high agreement between measurements (Intraclass correlation coefficient (ICC) at 0.97, 0.95 and 0.98 respectively). Despite very good agreement between the SCD test and TUNEL/FCM (ICC at 0.94), the SCD test tended to slightly but significantly underestimate DNA damage compared with TUNEL (*p* = 0.0127). This systematic difference between the two techniques was − 3.39 ± 1.45% (mean ± SE).

**Conclusions:**

Andrology laboratories using the SCD test to measure sperm DNA damage need to know that it appears to give slightly underestimated measurements compared to TUNEL/FCM. However, this systematic underestimation is very small in amplitude. Both techniques give almost perfectly congruent results. Our study underlines the importance for each laboratory to validate its method to assess sperm DNA damage before implementing it in routine andrology lab practice.

## Résumé

**Contexte:**

Plusieurs tests sont disponibles pour évaluer l’intégrité de l’ADN spermatique. Afin d’évaluer l’applicabilité de la technique de dispersion de la chromatine spermatique (SCD) dans un laboratoire d’andrologie, nous avons recruté 25 patients pris en charge au Centre de Médecine de la Reproduction du centre hospitalo-universitaire de Clermont-Ferrand (France). L’altération de l’ADN spermatique a été mesurée en ayant recours au test SCD et au test Terminal Uridine Nick-end Labeling en cytométrie en flux (TUNEL/CMF) dans les mêmes échantillons pour les deux techniques, après avoir réalisé un gradient de densité.

**Résultats:**

Pour le test SCD, la concordance entre les lectures, les lecteurs et les lames a été clairement établie avec un accord quasiment parfait entre les mesures (Coefficient de corrélation intra-classe (CCI) respectivement à 0,97, 0,95 et 0,98). Malgré une bonne concordance entre le test SCD et le test TUNEL/CMF (CCI à 0,94), le test SCD tend à sous-estimer légèrement mais de façon significative l’altération de l’ADN spermatique en comparaison avec le test TUNEL (*p* = 0,0127). Cette différence systématique entre les 2 techniques était de − 3.39 ± 1.45% (moyenne ± erreur standard).

**Conclusions:**

les laboratoires d’andrologie utilisant le test SCD pour mesurer l’altération de l’ADN spermatique doivent savoir qu’il donne apparemment des valeurs légèrement sous-estimées en comparaison du test TUNEL/CMF. Cependant, cette sous-estimation systématique est. de faible amplitude et les deux techniques donnent des résultats presque parfaitement concordants dans notre étude. Cette dernière montre bien que chaque laboratoire doit valider sa méthode sur site pour évaluer l’altération de l’ADN spermatique avant de le mettre en place en pratique quotidienne en andrologie.

## Background

Sperm DNA damage is an important semen quality parameter and a potential predictive biomarker of fertility [1–3]. Accurate determination of sperm DNA damage has important implications for assisted reproductive technology practice, but the lack of standardization is a bottleneck to routine use of sperm DNA integrity tests [4, 5]. Numerous tests have been proposed to evaluate sperm DNA integrity. Sperm DNA fragmentation can be measured by terminal uridine nick-end labeling (TUNEL), which remains a reference technique for direct measurement of DNA strand breaks [6–8]. Another reference technique is the Sperm Chromatin Structure Assay (SCSA). This latter measures two different sperm nuclear parameters: sperm DNA strand breaks and uncondensed chromatin [9]. The use of flow cytometry (FCM) to detect sperm with DNA fragmentation by TUNEL (or by SCSA) is considered a much more reliable technique than a slide-based analysis as it allows quick and easy automated measurement of a large number of spermatozoa. Moreover, it is clearly demonstrated that FCM is a sensitive, objective and precise method for detecting DNA fragmentation in spermatozoa [6, 10, 11]. However, it requires expensive instrumentation and is not easy to apply in routine analysis. Sperm chromatin dispersion (SCD) is an assay that measures the susceptibility of sperm to DNA denaturation [12]. After acid denaturation and nuclear protein removal, sperm without DNA fragmentation forms a halo whose diameter decreases with degree of DNA damage. SCD thus looks to be quick, easy and well-adapted to routine lab assessment of human sperm DNA fragmentation, but halo readings need an evaluation of intra and inter-observer reliability to validate their reproducibility.

Here we assessed the reliability of the SCD test coupled to bright-field microscopy and ran the very first comparison of the SCD test against the TUNEL assay with FCM for each sperm sample. The aim of the study was to determine whether the SCD test and TUNEL/FCM can be used interchangeably to measure DNA damage in routine andrology lab practice.

## Materials and methods

### Study design and procedures

**Fig. 1:**
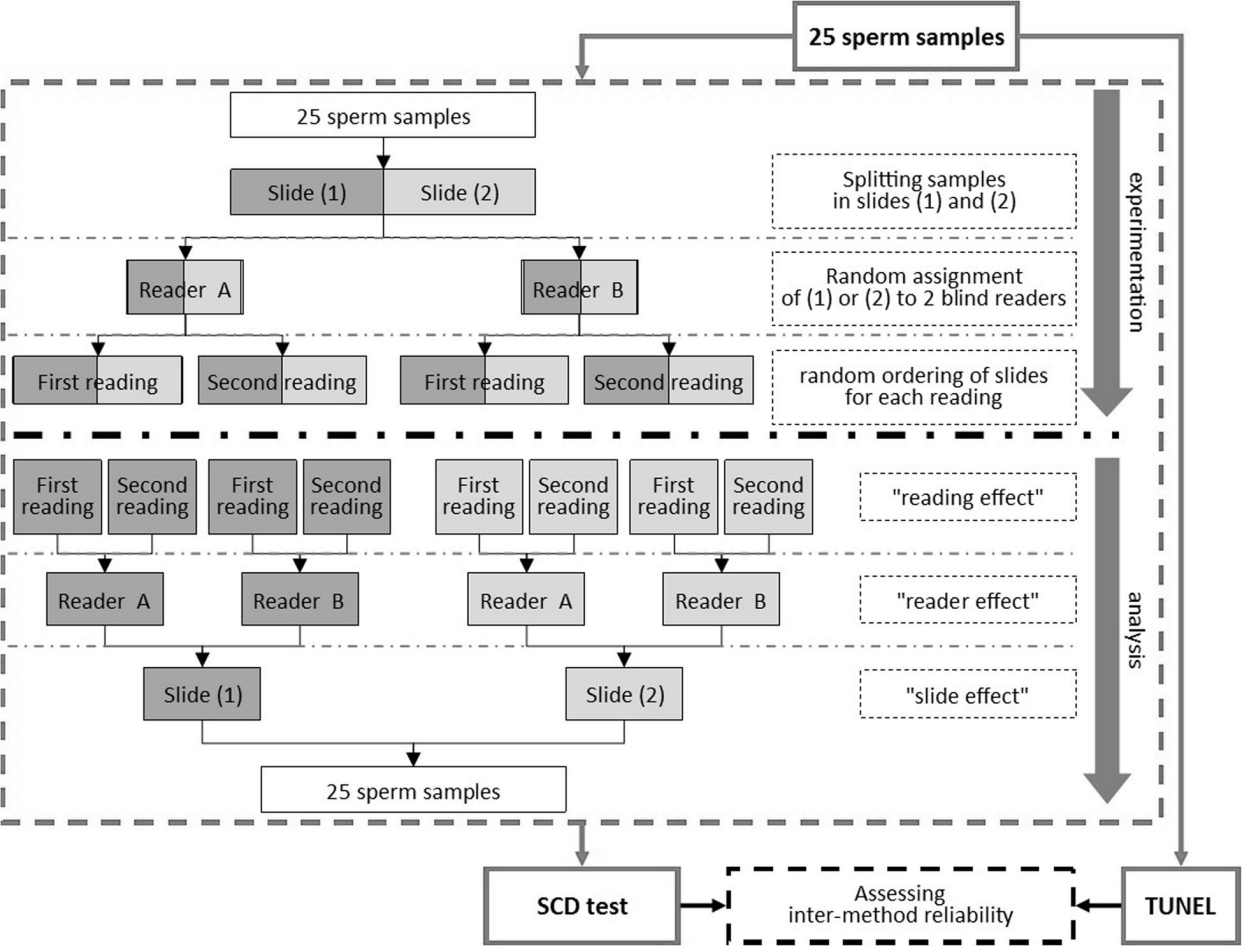
Study design. DNA damage was measured in sperm samples by both the SCD test and the TUNEL assay. For SCD test: the intra-observer, inter-observer and inter-slide reliabilities were successively assessed. Afterwards, the inter-method reliability between the SCD test and the TUNEL assay was assessed

The decision was made to evaluate the sperm DNA damage not on neat semen but after density gradient centrifugation. This should allow to get results within the potential available population of spermatozoa intended to be used in assisted reproductive techniques (ART). After density gradient centrifugation, sperm DNA damage was measured in sperm samples by both the SCD test and the TUNEL assay. Since the SCD test is a non-automated and subjective method, inter-slide reliability for readings of the same sperm sample and the intra- and inter-observer reliabilities of the same slide were assessed. Afterwards, we assessed the inter-method reliability between the SCD test and TUNEL/FCM for the measurements of sperm DNA damage (Fig. 1).

### Sperm collection and preparation

This study was performed on 25 men attending Clermont-Ferrand (France) University Hospital’s Center for Reproductive Medicine for fertility issues. All 25 patients attended the Center because of fertility issues, and no sperm donor was included. One semen sample was collected for each of them.

The andrology laboratory has implemented a quality management system based on the International Standard ISO 15189.

Semen samples were collected by masturbation into sterile containers after a period of 2–3 days of sexual abstinence. After semen liquefaction for 30 min at 37 °C, basic semen analysis was performed according to World Health Organization guidelines [13], except for sperm morphology assessment, which was done according to David morphological classification [14].

To isolate sperm cell populations, a two-step discontinuous Sperm Filter® (Cryo Bio System, Rambouillet, France) gradient (90–45%) diluted in Sperm Preparation Medium® (Origio, Limonest, France) was applied on the sperm samples. The purified sperm population was recovered from the 95% layer, washed in Sperm Prep Medium® (750 g, 8 min) and suspended in a suitable volume of phosphate buffer saline (PBS, Sigma-Aldrich, Lyon, France) supplemented with 1% (v/v) Bovine Serum Albumin (Sigma-Aldrich, Lyon, France) according to the manufacturer’s protocol for SCD tests (Halotech DNA, Spain) to reach a final concentration of 5 to 10.10^6^ spermatozoa.mL^− 1^.

### Sperm DNA damage

#### SCD test (Halosperm® kit)

The SCD test was performed using the Halosperm® kit based on the manufacturer’s protocol (Halotech DNA, Spain). The Eppendorf tubes of low-melting point agarose provided in the kit were placed in a water bath at 90–100 °C for 5 min. At the same time, the pre-coated slides were placed on a tray at 4 °C for 5 min. From this point, the protocol was applied twice in a row for each prepared sperm sample in order to get two slides in the end (50 slides for 25 sperm samples). The melted agarose was quickly added with 60 μL of each sperm sample and mixed. Sixty μL were thus pipetted twice in a row for each sperm sample and put in two different Eppendorf tubes. The chilled pre-coated slides were pipetted with 20 μL of the cellular suspensions, immediately covered (22 × 22 mm coverslip), then held at 4 °C for 5 min. Once the gel formed with the spermatozoa embedded inside, the coverslips were gently removed and the denaturation solution provided in the kit (containing hydrochloric acid) was applied for 7 min at room temperature. The slides were then placed in the lysing solution (Triton X-100, Dithiothreitol) for 25 min, and washed with distilled water for 5 min at room temperature. After dehydration by successive increasing concentrations of ethanol (70, 90 and 95%), the slides were dried and readied for bright-field microscopy by staining for 15 min with Wright staining solution (Merck 1.01383.0500, Darmstadt, Germany) and PBS (1:1, Merck 1.07294.1000, Darmstadt, Germany). These staining solutions are not provided in the kit, but are used by Fernández et al. [15]. The slides were mounted using Eukitt® mounting medium (O. Kindler GmbH & Co, Germany), a colorless medium with crystal-clear optics, which does not change color nor structure of mounted material (according to the technical data sheet). The slides were then stored in the dark at room temperature. This approach thus made it possible to take different readings at different times.

Positive controls were performed for each measurement. After incubation in permeabilization solution (0.1% sodium citrate, 0.1% Triton X-100) for 30 min at room temperature, spermatozoa were treated by DNAse I (Roche Diagnostics GmbH, Germany) at a final concentration of 3 IU.mL^− 1^ at 37 °C for 30 min and washed in PBS/BSA 1% (v/v) before measuring DNA damage by a SCD test as detailed above.

As described previously [15], the observed spermatozoa were scored in five patterns. A total 200 spermatozoa were scored per slide and per observer.

As the aim of the study was to assess the reliability of the SCD test, the study design (see below) was planned such that each sperm sample (once migrated) was split by preparing two slides. Each slide was read independently by two different blinded readers in random order. The coding of the slides had been done by a third person. Each reader ignored the value of the measure obtained by the other reader and each reader performed a double reading, also in random order. Slides had been re-coded before any reassessment by the same observer.

### TUNEL assay

The TUNEL assay was performed with flow cytometry as previously described before [16] to select the population of spermatozoa and to discard the debris and round cells. DNA fragmentation was detected with the “*in situ* cell death detection kit” according to the manufacturer’s protocol (Roche, Meylan, France). Briefly, 1.5 × 10^6^ washed spermatozoa were fixed with 2% paraformaldehyde for 30 min at room temperature. The spermatozoa were then rinsed and incubated for 3 min in permeabilization solution containing 0.1% Triton X-100 (v/v) in 0.1% citrate (w/v) on ice. After washing, the spermatozoa were labeled with 50 μL labeling solution containing dUTP and 50 μL terminal deoxynucleotidyl transferase (TdT). The incubation lasted 60 min at + 37 °C in a humidified atmosphere in the dark. After counterstaining with 2 mg.mL^− 1^ propidium iodide (PI), measurement was performed by flow cytometry.

For each sample, we ran a negative control by omitting the TdT enzyme and a positive control by incubating the spermatozoa with 3 IU DNase I for 15 min at 37 °C in Tris-HCl buffer before labeling. Flow cytometry was performed on an Epics XL cytometer (Beckman-Coulter, USA). A minimum of 20,000 spermatozoa were examined for each assay. Spermatozoa obtained in the plots of CMF were gated by using side-angle light scatter (SSC) and forward-angle light scatter (FSC). This was done to put out of the gate, debris and other cells than spermatozoa. An additional figure gives more details about flow cytometry measurements (see Additional file 1).

An argon laser delivered a 488 nm excitation wavelength. Green fluorescence (TUNEL-positive cells) was detected with FL1 (using a 525-nm band-pass filter) and red fluorescence (PI-positive cells) with FL3 (using a 620-nm band-pass filter). Both fluorescence signals were recorded after logarithmic amplification. Rate of labeled cells was calculated by the flow cytometer software.

### Statistical analyses

**Fig. 2:**
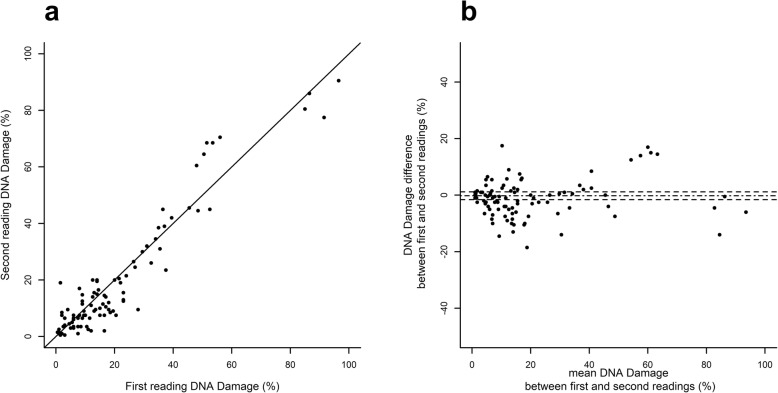
“Reading effect” for SCD test. Scatterplot (**a**) of second vs first reading by the same reader and Bland-Altman plot (**b**) where the difference (second reading minus first reading) is plotted against the mean (arithmetic mean of two readings of each slide), the mean difference is shown as a dash-dot line and its 95% confidence limits are shown as two dashed lines

**Fig. 3:**
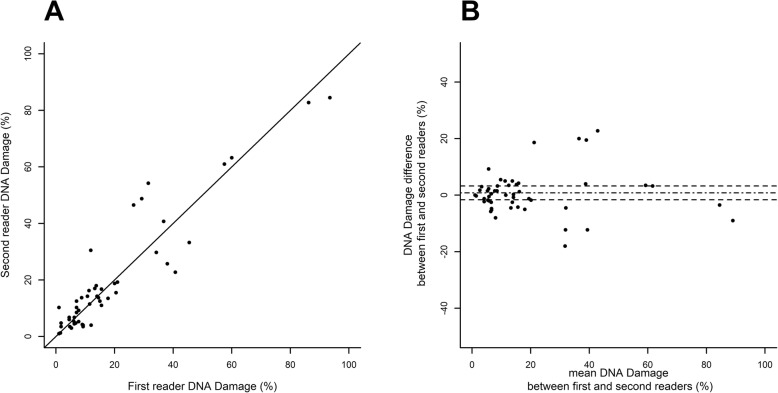
“Reader effect” for SCD test. Scatterplot (**a**) of second reader vs first reader of the same slide and Bland-Altman plot (**b**) where the difference (second reader minus first reader) is plotted against the mean (arithmetic mean of two readers for each slide), the mean difference is shown as a dash-dot line and its 95% confidence limits are shown as two dashed lines

**Fig. 4:**
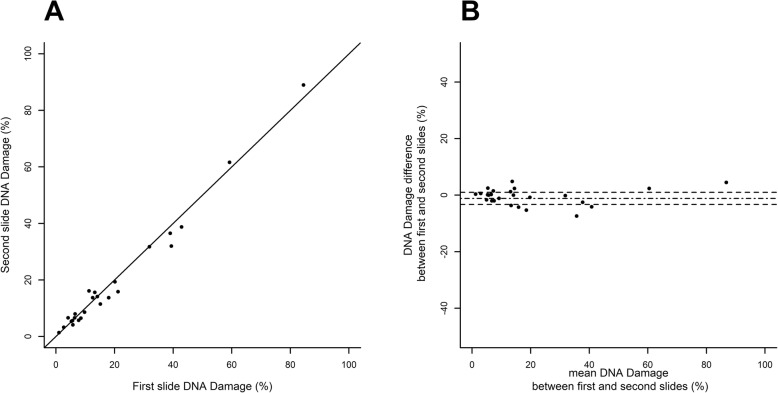
“Slide effect” for SCD test. Scatterplot (**a**) of second slide vs first slide of the same sperm sample and Bland-Altman plot (**b**) where the difference (second slide minus first slide) is plotted against the mean (arithmetic mean of two slides for each sperm sample), the mean difference is shown as a dash-dot line and its 95% confidence limits are shown as two dashed lines

All analyses are based on the same sperm samples from 25 patients. The first focus of the study was the reliability of SCD test in measuring sperm DNA damage. We tested for the following potential factor effects: the effect of preparing several slides from the same sperm sample (referred to as “slide effect”), the effect of involving several readers for the same slide (referred to as “reader effect”), and the effect of one reader reading the same slide several times (referred to as “reading effect”). Thus, regarding the SCD test, each sperm sample was split into two slides, each slide was read by two readers (the same pair of trained observers for the whole study), and each reader read each slide twice. The reliability of SCD was assessed using a hierarchical frame following the same scheme for each factor. First, the factor effect was tested through a discordance test using a paired Student *t*-test or a non-parametric signed-rank test if differences showed non-normal distribution (assessed by a Shapiro–Wilk test). When the tests found no significant discordance on a factor, the concordance between the two modalities of this factor was estimated using the intraclass correlation coefficient (ICC) [17]. In cases of non-discordant values and very good to almost perfect concordance (ICC at 0.8 or more), the two available values were lumped together by computing their mean. Once a factor was assessed, analyses moved on to focus on the next factor, following the same scheme. Analyses followed a hierarchical schedule, first testing the “reading effect”, then the “reader effect” and finally the “slide effect”, according to the average differences which were expected to sort in ascending order from difference between readings (see Fig. 2), then between readers (see Fig. 3), and lastly between slides (see Fig. 4). Scatterplots and Bland–Altman plots were graphed for each factor analysis [18] (see Figs. 2 to 4).

If, as expected, the quantifications of DNA damage measured by SCD were sufficiently reliable and reproducible, inter-method reliability between SCD and TUNEL was assessed following the same experimental design in these same 25 patients. Finally, we assessed the relationship between sperm parameters and DNA damage (through both SCD test and TUNEL assay) by performing non-parametric Spearman correlation coefficient tests.

All statistical analyses were performed using SAS v9.4 for windows (SAS Institute Inc., Cary, NC) with a double-sided type I error set at 0.05.

## Results

### Patients and semen characteristics

Main results are presented in Table 1.

**Table 1:** Patients and semen characteristics

Variable	N	Mean	Median	Std Dev^a^	Minimum	Maximum	Lower Quartile	Upper Quartile
Age (years)	25	38.60	38.0	6.06	30.0	55.0	35.0	40.0
Sperm concentration (million/mL)	25	110.04	80.0	102.24	15.0	432.0	41.0	150.0
Total sperm number (million)	25	395.39	243.6	379.85	57.5	1530.0	161.5	567.3
Progressive motility (%)	25	39.40	40.0	14.33	13.0	70.0	30.0	49.0
Total motility (%)	25	47.92	50.0	14.33	23.0	82.0	37.0	55.0
Initial vitality (%)	25	74.56	74.0	9.47	53.0	88.0	67.0	82.0
Initial normal sperm morphology (%)	25	15.70	16.0	8.48	1.0	31.0	11.0	21.0
Normal sperm morphology after sperm preparation (%)	25	24.39	25.0	11.56	2.0	43.0	18.0	34.0

^a^Std Dev: Standard deviation. Sperm motility was analyzed by measurements of progressive motility and total motility as it is mentioned in WHO’s guidelines

Intra-method (SCD) and inter-method (SCD versus TUNEL) reliability analyses

The key results on the reliability analyses are reported in Table 2. Full data are available in Additional files 2 and 3.

**Table 2:** Assessment of reliability of DNA damage (in percentages)

Reliability analysis	Effect	N	Mean difference (SE)	*p*-value*	ICC^a^
Within SCD	Reading	200	− 0.205 (0.70)	0.3975	0.96632
	Reader	100	0.816 (1.25)	0.8213	0.94680
	Slide	50	−1.142 (1.08)	0.5195	0.98251
Between SCD and TUNEL	Technique	50	−3.392 (1.45)	0.0127	0.93834

Reliability analysis within SCD test and inter-method reliability between the SCD test and the TUNEL assay

*p-value of the signed-rank test

^a^*ICC* Intraclass Correlation Coefficient

### Reliability assessment for SCD test measurement of DNA damage

The reliability of the SCD test in measuring sperm DNA damage was assessed by analyzing the “reading”, “reader” and “slide” effects. For each of the 25 sperm samples, two slides were prepared and each slide was read twice by each reader. This experiment involved two observers, reading the slides independently and in random order.

“Reading” effect was assessed within 200 readings. Mean difference between the first and second reading of the same slide by the same reader was − 0.20 ± 0.70% (mean ± SE) and was not significantly different from 0 (*p* = 0.3975). ICC was 0.97, reflecting an almost perfect agreement of SCD test measures between readings (see the scatterplot and Bland–Altman plot in Fig. 2).

“Reader” effect was assessed within 100 readings, pooling both readings by the same reader by their mean since there was almost perfect reading-to-reading agreement. Mean difference between readers of the same slide was 0.82 ± 1.25% (mean ± SE) and was not significantly different from 0 (*p* = 0.8213). ICC was 0.95, reflecting a very good agreement of SCD test measures between readers (see the scatterplot and Bland –Altman plot in Fig. 3).

“Slide” effect was assessed within 50 readings, pooling measures from both readers of the same slide by their mean since there was very good reader-to-reader agreement. Mean difference between slides of the same sperm sample was − 1.14 ± 1.08% (mean ± SE) and was again not significantly different from 0 (*p* = 0.5195). ICC was 0.98, reflecting an almost perfect agreement of SCD test measures between two slides from the same sperm sample (see the scatterplot and Bland–Altman plot in Fig. 4).

SCD test measurements from both slides of the same sperm sample were lumped together by their mean since there was almost perfect slide-to-slide agreement, leading to 25 measurements of DNA damage from SCD tests.

### Inter-method reliability between the SCD test and TUNEL

**Fig. 5:**
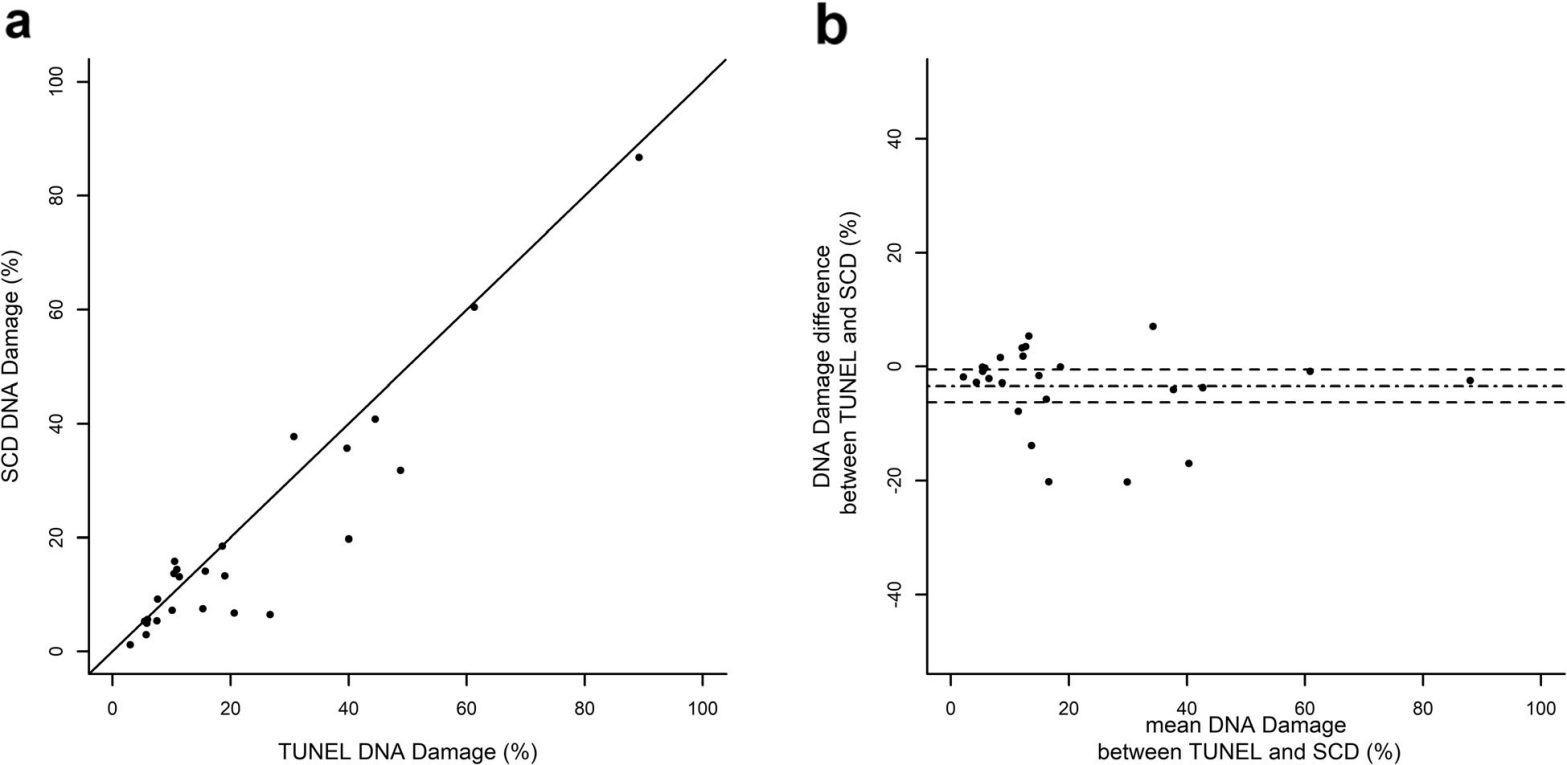
Scatterplot (**a**) of SCD vs TUNEL and Bland-Altman plot (**b**). The difference for DNA damage (SCD minus TUNEL) is plotted against the mean, the mean difference is shown as a dash-dot line and its 95% confidence limits are shown as two dashed lines

The inter-method reliability for measuring sperm DNA damage was assessed within 50 readings since each sperm sample (*n* = 25) was first split in two to perform each DNA damage measurement. As shown in Fig. 5, DNA damage exhibited distribution with quite a wide range of values, for both TUNEL/FCM and SCD. The mean ± SE value of DNA damage was 22.6 ± 4.2% for TUNEL/FCM and 19.2 ± 4.0% for SCD. DNA damage ranged from 3 to 89.2% for TUNEL/FCM and from 1.2 to 86.8% for SCD. The median (and interquartile) limits were 15.3% ([7.6–30.7]) for TUNEL/FCM and 12.5% ([6.4–20.1]) for SCD. Mean difference between methods of the same sperm sample was − 3.39 ± 1.45% (mean ± SE) and turned out to be significantly different from 0 (*p* = 0.0127). Nevertheless, as shown by scatterplot and Bland–Altman plot (Fig. 5), DNA damage measurements were very close to each other. Compared to TUNEL, SCD tends to underestimate DNA-damage with a systematic offset of about 3.4%.

Inter-method ICC was 0.94, meaning that despite a systematic offset of − 3.39%, the results from these two methods can be considered very highly concordant.

### Correlation between sperm DNA damage and standard semen parameters

Significant negative correlations between sperm DNA damage (using both SCD test and TUNEL assay results) and sperm characteristics were found for progressive motility, total motility, vitality, and initial morphology. No significant correlation was observed between sperm DNA damage and total sperm number, sperm concentration, or sperm morphology after sperm preparation.

The results on the correlation analyses are reported in Table 3.

**Table 3:** Correlations between sperm DNA damage and sperm characteristics

Sperm characteristics (*N* = 25)	SCD		TUNEL	
	SCC^a^	*p*-value	SCC^a^	*p*-value
Total sperm number	0.12615	0.5479	−0.00385	0.9854
Sperm concentration	0.06193	0.7687	−0.05732	0.7855
Progressive motility	−0.44795	0.0247	−0.47489	0.0164
Total motility	−0.60312	0.0014	−0.64934	0.0004
Sperm morphology after sperm preparation	−0.31332	0.1272	−0.35296	0.0835
Initial sperm morphology	−0.42040	0.0364	−0.57324	0.0027
Initial sperm vitality	−0.43100	0.0315	−0.43793	0.0286

^a^*SCC* Spearman Correlation Coefficient

## Discussion

Sperm DNA damage can result from defective chromatin packaging [15], abortive apoptosis [19, 20] or oxidative stress [21]. A high level of sperm DNA damage negatively influences live birth rate [22]. Since sperm DNA integrity is an important component of fertility, andrology labs need an accurate method for measuring sperm DNA integrity. Sperm Chromatin Dispersion (SCD) test and the TUNEL assay are two available methods to measure sperm DNA damages. The TUNEL assay with flow cytometry detection is considered as the reference method for detecting DNA breaks [23] but does not lend itself to easy routine practice. Here we clearly showed that in our laboratory the SCD test with bright microscopy is highly reliable, accurate and does not require andrology labs to invest in expensive new instrumentation.

Since the SCD test is a non-automated method, we first analyzed the potential subjectivity in measurements by a blinded experiment with two different readers. Our results clearly showed high reliability between readings of the same reader, between readers of the same slide, and even between slides of the same sperm sample. Our results are in accordance with a previous study [15] showing very low within-reader and between-reader variability in 6 readings of 4 different readers. However, it is important to note that the readers have to be trained to perform the measurements. Indeed, it was not always easy to distinguish the difference between class 2 and 3 spermatozoa described by Fernández [15] using the SCD test. Mounted slides with known SCD test results should be kept as a reference to enable regular training of expert readers and ensure high inter-reader reliability. Furthermore, each andrology lab needs to optimize its staining conditions as it is crucial to easily distinguish the halo from the core. The optimal staining, i.e. the time required to get an easily distinct halo of dispersed DNA loop, has to be found in each lab. The halo must not be too dim, which would risk making the outer edge hard to see, nor too intense, which would risk making the borderline between halo and core difficult to determine. Using a microscope eyepiece reticle could help distinguish the different classes of observed spermatozoa.

Our analysis showing high reliability between two slides from the same sperm sample brings novel findings and underlines that the SCD test is a robust measure despite the fact that this technique is non-automated.

The inter-method analysis performed here revealed high agreement between results from the SCD test and the flow-cytometry TUNEL assay. This is the first study to thoroughly and correctly compare these two methods (SCD vs TUNEL/FCM) based on measurements performed in the same sperm samples. It is above all an objective technical comparison between two techniques of evaluation of sperm DNA damage, SCD versus TUNEL/FCM. Another study reported a high correlation between these two techniques [24] in frozen sperm samples, but failed to perform a reliability analysis. Another one [25] showed a higher level of sperm DNA damage when measurements were performed with SCD test compared to the TUNEL assay with detection by fluorescence microscopy (FM). This is not in accordance with our results but limitations of FM are well-known. Indeed a few hundred cells are observed by this method with the risk of fluorescence bleaching during analysis and relying on human eye. Evenson et al. [26] brought interesting remarks to the article of Fernández et al. [15], enhancing the power of flow cytometry (with the SCSA test) versus the optic microscopy. With the detection by FCM, the gating of the population of interest in the dot plots warrants special care. In this study we followed previous gating protocols as published by Grizard et al. [27]. Some authors working on TUNEL/FCM technique excluded semen samples with considerable leukocytospermia [11, 28]. The selection by density gradient centrifugation (DGC) removed the major part of round cells, leukocytes and debris, which made the TUNEL/FCM technique easier to carry out and more reliable. The sperm selection methods such as DGC are known to improve general sperm parameters and to reduce sperm DNA fragmentation [29]. However, the improvement of sperm DNA integrity may not be as important as the improvement of sperm motility [30–32]. As expected, in our study, sperm DNA damage in sperm suspensions after DGC, assessed with both TUNEL/FCM and SCD techniques exhibited distribution with a wide range of values. Working on prepared samples gave supplementary work for this study, but we thought it was important to do that way, keeping in mind that if the results finally led to the choice of SCD test as a routine technique, the procedure would directly be validated on prepared samples. A further clinical study would possibly be conducted in a second time on intra-uterine insemination and in-vitro fertilization cycles. This perspective could be helpful to define a cut-off value, predictive of clinical pregnancies and live birth rates after ART.

Despite high reliability between SCD test and flow-cytometry TUNEL assay results from the same sperm sample, a systematic higher proportion of sperm DNA damage was observed by TUNEL. This systematic offset may be explained by lower detection sensitivity (eye-dependent), fewer cells analyzed, and differences in the principles underpinning these two methods. Indeed, the SCD test measures the susceptibility of DNA to acid denaturation [12] while the TUNEL assay measures DNA fragmentation by incorporation of modified nucleotides (dUTP) at the site of DNA damage [33, 34]. Fernandez et al. [12] showed that when spermatozoa are not exposed to a denaturing acid solution, it is difficult to distinguish differences in nuclear DNA dispersion between spermatozoa with fragmented and nonfragmented DNA. In contrast, when sperm with DNA fragmentation are exposed to a denaturing acid solution prior to deproteinization, the halos of DNA dispersion are absent or extremely small compared to those observed in sperm nuclei with no DNA fragmentation. In that study it was also showed that the rate of single strand DNA (ssDNA) increases after a denaturing step, in case of DNA breaks. However, the suppression of the production of DNA halos in sperm nuclei with extensive DNA fragmentation remains not well understood. It may be possible that no loop can appear in case of sperm DNA fragmentation due to interactions between the ssDNA and sperm head after the removal of proteins by the lysing solution. This systematic difference confirms the lack of correlation showed by a previous study [35] reporting differences in quantified DNA damage, and implies that only results measured by either the SCD test or the TUNEL should be cross-compared.

In our setting, the rapid solidification of the agarose for the SCD test could be a drawback when testing numerous sperm samples in parallel. We determined that a maximum of 3 tests could be performed together. As the Halosperm® kit is not compatible for fixed samples; we performed the test on only fresh sperm samples, though simultaneous tests would become possible if the samples were thawed at the same time. Our fresh-only protocol ensured that the study was not affected by the potential effect of cryopreservation on DNA damage [36]. Flow cytometry TUNEL is able to measure a higher number of samples, but the assay is also time-consuming and requires an expensive flow cytometer. For some laboratories with a high level of activity, an option would be to buy a used and refurbished flow cytometer to apply the TUNEL assay. We used positive controls to validate our assays but it enhanced the time of measurements. A good option could be to use positive controls frozen in advance. The SCD test is a quick and easy technique to implement in routine practice (1.5 h), and in contrast to flow-cytometry TUNEL (around 4 h for a series of samples, not for a single one), it can also be adapted to low-spermatozoa-count sperm samples.

When taking into account all cost parameters and the feasibility in routine andrology lab settings, the SCD test emerges as a more suitable option than the flow-cytometry TUNEL assay although the cost of one test for the commercial kit of the SCD test was higher than for TUNEL assay commercial kit. This is mainly explained by the expensive investment for acquisition and maintenance of flow cytometer.

Both the SCD test and the TUNEL assay pointed to statistically significant negative correlations between sperm DNA damage and sperm motility and morphology. This is consistent with previous studies [37–39] and confirms that male infertility is associated with poor sperm DNA integrity. The lack of negative correlation with sperm concentration may be explained by the high initial sperm concentration required to perform our numerous reliability analyses.

## Conclusion

In conclusion, the SCD test offers a practicable and reliable option. Our study showed that despite a systematic offset of 3.39%, results from the SCD test and from TUNEL/FCM can be considered almost perfectly concordant. Andrology labs need to look carefully at which technique to use to evaluate sperm DNA damage. Very strict procedures must be followed and consistent intra- and inter-laboratory validation should be made. It could be very interesting, if possible, to compare the results to a laboratory using cytometric assays (TUNEL or SCSA), but it is not always available. In many countries around the world, at least one national organization is responsible for the accreditation of the country’s medical laboratories. The ISO 15189 is a uniform approach to evaluate a laboratory competence. It is implemented in andrology laboratories in numerous countries and also in France. It has helped laboratories to adopt internationally accepted measurement practices. Before implementing SCD test, it is necessary to validate it by each andrology laboratory, as performed in this study. By determining ICC for readings, readers and slides, and through all the quality process in the laboratory, the technical competence of the staff and the validity and the appropriateness of the method can be ensured.

External quality controls exist for the standard sperm parameters (sperm output, motility and morphology) but none were found for sperm DNA damages tests. This is a real weakness for the standardization of these tests and reduces the scope of clinical studies proposing clinical cut-off values.

## Supplementary information

**Additional file 1.** Flow cytometry charts. This figure shows the charts of CMF for the patient number one and its positive control.

**Additional file 2.** Variable dictionary. This table gives the explanation of the variables used in Additional file 3.

**Additional file 3.** Exhaustive results of sperm DNA damage. This table gives the full data of the DNA fragmentation results for each patient, slide, reader, reading and method.

## Data Availability

All data generated or analyzed during this study are included in this published article and its additional information files.

## Abbreviations

%: Percentage
ART: Assisted reproductive techniques
DGC: Density gradient centrifugation
dif: Difference
DNase: Deoxyribonuclease
dUTP: Deoxyuridine 5-triphosphate
FCM: Flow cytometry
FSC: Forward-angle light scatter
ICC: Intraclass correlation coefficient
ISO: International organization for standardization
lab: Laboratory
min: Minute
SCC: Spearman correlation coefficient
SCD: Sperm chromatin dispersion
SCSA: Sperm chromatin structure assay
SE: Standard error
SSC: Side-angle light scatter
ssDNA: Single strand dnA
Std Dev: Standard deviation
TdT: Terminal deoxynucleotidyl transferase
TUNEL: Terminal uridine nick-end labeling
vs: Versus
WHO: World health organization
