# Supplementary material for: Can the SCD test and terminal uridine nick-end labeling by flow cytometry technique (TUNEL/FCM) be used interchangeably to measure sperm DNA damage in routine laboratory practice?
Source: Basic Clin Androl. 2019 Dec 26;29:17. doi: 10.1186/s12610-019-0098-2 (PMC6933933; doi:10.1186/s12610-019-0098-2)
Supplement: Supplementary file 1 — Additional file 1. Flow cytometry charts. This figure shows the charts of CMF for the patient number one and its positive control. [file 12610_2019_98_MOESM1_ESM.doc]

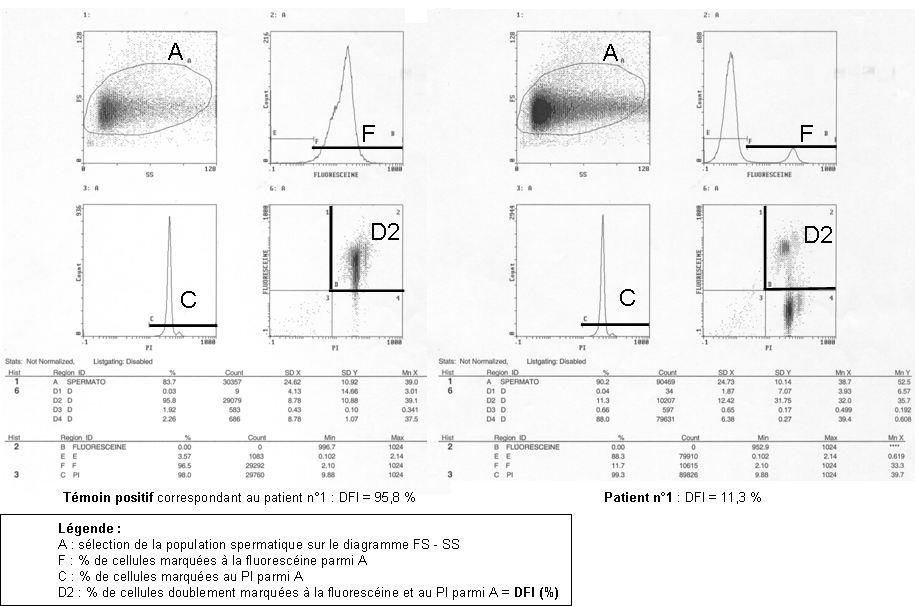


**Legend :**

A: sperm cells gated on a forward scatter (FS)/side scatter (SS) plot

F: cells labeled with fluorescein among A selection (%)

C: cells labeled with PI among A selection (%)

D2: cells labeled with fluorescein and PI among A selection = DFI (%)

**Patient n°1:** DFI = 11.3 %

**Positive control** for patient n°1: DFI = 95.8 %
