# Supplementary material for: Can the SCD test and terminal uridine nick-end labeling by flow cytometry technique (TUNEL/FCM) be used interchangeably to measure sperm DNA damage in routine laboratory practice?
Source: Basic Clin Androl. 2019 Dec 26;29:17. doi: 10.1186/s12610-019-0098-2 (PMC6933933; doi:10.1186/s12610-019-0098-2)
Supplement: Supplementary file 2 — Additional file 2. Variable dictionary. This table gives the explanation of the variables used in Additional file 3. [file 12610_2019_98_MOESM2_ESM.doc]

**S1 Table. Variable dictionary.**

| VARIABLE DICTIONARY | | |
| --- | --- | --- |
| DATASET | VARNAME | CONTENT |
| PC_FRAG | patient | anonymous number : 1 to 25 (linkage key) |
|  | method | method : 1 = SCD, 0 = TUNEL |
|  | slide | slide number (for SCD method only) |
|  | reader | reader number (for SCD method only) |
|  | reading | reading number (for SCD method only) |
|  | pc_frag | Sperm DNA damage (%) |
