# Supplementary material for: Can the SCD test and terminal uridine nick-end labeling by flow cytometry technique (TUNEL/FCM) be used interchangeably to measure sperm DNA damage in routine laboratory practice?
Source: Basic Clin Androl. 2019 Dec 26;29:17. doi: 10.1186/s12610-019-0098-2 (PMC6933933; doi:10.1186/s12610-019-0098-2)
Supplement: Supplementary file 3 — Additional file 3. Exhaustive results of sperm DNA damage. This table gives the full data of the DNA fragmentation results for each patient, slide, reader, reading and method. [file 12610_2019_98_MOESM3_ESM.doc]

**S2 Table. Exhaustive results of sperm DNA damage.**

| PC_FRAG | | | | | |
| --- | --- | --- | --- | --- | --- |
| patient | method | slide | reader | reading | pc_frag |
| 1 | 0 | . | . | . | 11,3 |
| 1 | 1 | 1 | 1 | 1 | 9,0 |
| 1 | 1 | 1 | 1 | 2 | 12,5 |
| 1 | 1 | 1 | 2 | 1 | 13,0 |
| 1 | 1 | 1 | 2 | 2 | 15,5 |
| 1 | 1 | 2 | 1 | 1 | 18,0 |
| 1 | 1 | 2 | 1 | 2 | 12,0 |
| 1 | 1 | 2 | 2 | 1 | 15,0 |
| 1 | 1 | 2 | 2 | 2 | 10,0 |
| 2 | 0 | . | . | . | 7,5 |
| 2 | 1 | 1 | 1 | 1 | 5,5 |
| 2 | 1 | 1 | 1 | 2 | 5,0 |
| 2 | 1 | 1 | 2 | 1 | 2,5 |
| 2 | 1 | 1 | 2 | 2 | 3,5 |
| 2 | 1 | 2 | 1 | 1 | 16,5 |
| 2 | 1 | 2 | 1 | 2 | 2,0 |
| 2 | 1 | 2 | 2 | 1 | 5,0 |
| 2 | 1 | 2 | 2 | 2 | 3,0 |
| 3 | 0 | . | . | . | 15,3 |
| 3 | 1 | 1 | 1 | 1 | 9,0 |
| 3 | 1 | 1 | 1 | 2 | 6,5 |
| 3 | 1 | 1 | 2 | 1 | 9,5 |
| 3 | 1 | 1 | 2 | 2 | 9,0 |
| 3 | 1 | 2 | 1 | 1 | 6,0 |
| 3 | 1 | 2 | 1 | 2 | 6,5 |
| 3 | 1 | 2 | 2 | 1 | 6,0 |
| 3 | 1 | 2 | 2 | 2 | 7,5 |
| 4 | 0 | . | . | . | 20,6 |
| 4 | 1 | 1 | 1 | 1 | 7,5 |
| 4 | 1 | 1 | 1 | 2 | 6,5 |
| 4 | 1 | 1 | 2 | 1 | 9,5 |
| 4 | 1 | 1 | 2 | 2 | 7,5 |
| 4 | 1 | 2 | 1 | 1 | 11,0 |
| 4 | 1 | 2 | 1 | 2 | 2,5 |
| 4 | 1 | 2 | 2 | 1 | 6,0 |
| 4 | 1 | 2 | 2 | 2 | 3,5 |
| 5 | 0 | . | . | . | 7,6 |
| 5 | 1 | 1 | 1 | 1 | 10,5 |
| 5 | 1 | 1 | 1 | 2 | 3,5 |
| 5 | 1 | 1 | 2 | 1 | 8,0 |
| 5 | 1 | 1 | 2 | 2 | 17,0 |
| 5 | 1 | 2 | 1 | 1 | 12,0 |
| 5 | 1 | 2 | 1 | 2 | 2,0 |
| 5 | 1 | 2 | 2 | 1 | 1,5 |
| 5 | 1 | 2 | 2 | 2 | 19,0 |
| 6 | 0 | . | . | . | 10,4 |
| 6 | 1 | 1 | 1 | 1 | 10,0 |
| 6 | 1 | 1 | 1 | 2 | 7,5 |
| 6 | 1 | 1 | 2 | 1 | 16,0 |
| 6 | 1 | 1 | 2 | 2 | 11,5 |
| 6 | 1 | 2 | 1 | 1 | 16,5 |
| 6 | 1 | 2 | 1 | 2 | 14,5 |
| 6 | 1 | 2 | 2 | 1 | 14,0 |
| 6 | 1 | 2 | 2 | 2 | 19,5 |
| 7 | 0 | . | . | . | 5,9 |
| 7 | 1 | 1 | 1 | 1 | 4,5 |
| 7 | 1 | 1 | 1 | 2 | 4,5 |
| 7 | 1 | 1 | 2 | 1 | 4,0 |
| 7 | 1 | 1 | 2 | 2 | 9,5 |
| 7 | 1 | 2 | 1 | 1 | 1,0 |
| 7 | 1 | 2 | 1 | 2 | 1,0 |
| 7 | 1 | 2 | 2 | 1 | 9,0 |
| 7 | 1 | 2 | 2 | 2 | 11,5 |
| 8 | 0 | . | . | . | 18,6 |
| 8 | 1 | 1 | 1 | 1 | 9,0 |
| 8 | 1 | 1 | 1 | 2 | 14,8 |
| 8 | 1 | 1 | 2 | 1 | 37,5 |
| 8 | 1 | 1 | 2 | 2 | 23,5 |
| 8 | 1 | 2 | 1 | 1 | 17,0 |
| 8 | 1 | 2 | 1 | 2 | 10,5 |
| 8 | 1 | 2 | 2 | 1 | 23,0 |
| 8 | 1 | 2 | 2 | 2 | 13,0 |
| 9 | 0 | . | . | . | 39,7 |
| 9 | 1 | 1 | 1 | 1 | 45,5 |
| 9 | 1 | 1 | 1 | 2 | 45,5 |
| 9 | 1 | 1 | 2 | 1 | 35,5 |
| 9 | 1 | 1 | 2 | 2 | 31,0 |
| 9 | 1 | 2 | 1 | 1 | 34,0 |
| 9 | 1 | 2 | 1 | 2 | 34,5 |
| 9 | 1 | 2 | 2 | 1 | 29,5 |
| 9 | 1 | 2 | 2 | 2 | 30,0 |
| 10 | 0 | . | . | . | 89,2 |
| 10 | 1 | 1 | 1 | 1 | 86,5 |
| 10 | 1 | 1 | 1 | 2 | 86,0 |
| 10 | 1 | 1 | 2 | 1 | 85,0 |
| 10 | 1 | 1 | 2 | 2 | 80,5 |
| 10 | 1 | 2 | 1 | 1 | 96,5 |
| 10 | 1 | 2 | 1 | 2 | 90,5 |
| 10 | 1 | 2 | 2 | 1 | 91,5 |
| 10 | 1 | 2 | 2 | 2 | 77,5 |
| 11 | 0 | . | . | . | 5,8 |
| 11 | 1 | 1 | 1 | 1 | 6,0 |
| 11 | 1 | 1 | 1 | 2 | 6,0 |
| 11 | 1 | 1 | 2 | 1 | 7,5 |
| 11 | 1 | 1 | 2 | 2 | 3,5 |
| 11 | 1 | 2 | 1 | 1 | 3,0 |
| 11 | 1 | 2 | 1 | 2 | 6,5 |
| 11 | 1 | 2 | 2 | 1 | 3,0 |
| 11 | 1 | 2 | 2 | 2 | 4,0 |
| 12 | 0 | . | . | . | 48,8 |
| 12 | 1 | 1 | 1 | 1 | 37,0 |
| 12 | 1 | 1 | 1 | 2 | 39,0 |
| 12 | 1 | 1 | 2 | 1 | 27,0 |
| 12 | 1 | 1 | 2 | 2 | 24,5 |
| 12 | 1 | 2 | 1 | 1 | 36,5 |
| 12 | 1 | 2 | 1 | 2 | 45,0 |
| 12 | 1 | 2 | 2 | 1 | 24,0 |
| 12 | 1 | 2 | 2 | 2 | 21,5 |
| 13 | 0 | . | . | . | 44,5 |
| 13 | 1 | 1 | 1 | 1 | 31,0 |
| 13 | 1 | 1 | 1 | 2 | 32,0 |
| 13 | 1 | 1 | 2 | 1 | 48,0 |
| 13 | 1 | 1 | 2 | 2 | 60,5 |
| 13 | 1 | 2 | 1 | 1 | 35,0 |
| 13 | 1 | 2 | 1 | 2 | 38,5 |
| 13 | 1 | 2 | 2 | 1 | 39,5 |
| 13 | 1 | 2 | 2 | 2 | 42,0 |
| 14 | 0 | . | . | . | 40,0 |
| 14 | 1 | 1 | 1 | 1 | 21,5 |
| 14 | 1 | 1 | 1 | 2 | 20,5 |
| 14 | 1 | 1 | 2 | 1 | 23,0 |
| 14 | 1 | 1 | 2 | 2 | 15,5 |
| 14 | 1 | 2 | 1 | 1 | 20,0 |
| 14 | 1 | 2 | 1 | 2 | 20,0 |
| 14 | 1 | 2 | 2 | 1 | 28,0 |
| 14 | 1 | 2 | 2 | 2 | 9,5 |
| 15 | 0 | . | . | . | 10,9 |
| 15 | 1 | 1 | 1 | 1 | 17,0 |
| 15 | 1 | 1 | 1 | 2 | 14,0 |
| 15 | 1 | 1 | 2 | 1 | 13,0 |
| 15 | 1 | 1 | 2 | 2 | 9,0 |
| 15 | 1 | 2 | 1 | 1 | 23,0 |
| 15 | 1 | 2 | 1 | 2 | 12,5 |
| 15 | 1 | 2 | 2 | 1 | 18,5 |
| 15 | 1 | 2 | 2 | 2 | 8,5 |
| 16 | 0 | . | . | . | 10,5 |
| 16 | 1 | 1 | 1 | 1 | 22,0 |
| 16 | 1 | 1 | 1 | 2 | 19,0 |
| 16 | 1 | 1 | 2 | 1 | 14,5 |
| 16 | 1 | 1 | 2 | 2 | 16,5 |
| 16 | 1 | 2 | 1 | 1 | 15,0 |
| 16 | 1 | 2 | 1 | 2 | 7,5 |
| 16 | 1 | 2 | 2 | 1 | 12,5 |
| 16 | 1 | 2 | 2 | 2 | 20,0 |
| 17 | 0 | . | . | . | 26,7 |
| 17 | 1 | 1 | 1 | 1 | 9,5 |
| 17 | 1 | 1 | 1 | 2 | 9,0 |
| 17 | 1 | 1 | 2 | 1 | 3,0 |
| 17 | 1 | 1 | 2 | 2 | 4,0 |
| 17 | 1 | 2 | 1 | 1 | 11,5 |
| 17 | 1 | 2 | 1 | 2 | 6,5 |
| 17 | 1 | 2 | 2 | 1 | 7,5 |
| 17 | 1 | 2 | 2 | 2 | 1,0 |
| 18 | 0 | . | . | . | 5,4 |
| 18 | 1 | 1 | 1 | 1 | 4,5 |
| 18 | 1 | 1 | 1 | 2 | 4,5 |
| 18 | 1 | 1 | 2 | 1 | 8,5 |
| 18 | 1 | 1 | 2 | 2 | 3,5 |
| 18 | 1 | 2 | 1 | 1 | 6,0 |
| 18 | 1 | 2 | 1 | 2 | 6,5 |
| 18 | 1 | 2 | 2 | 1 | 6,0 |
| 18 | 1 | 2 | 2 | 2 | 3,0 |
| 19 | 0 | . | . | . | 15,7 |
| 19 | 1 | 1 | 1 | 1 | 14,0 |
| 19 | 1 | 1 | 1 | 2 | 15,0 |
| 19 | 1 | 1 | 2 | 1 | 18,0 |
| 19 | 1 | 1 | 2 | 2 | 9,5 |
| 19 | 1 | 2 | 1 | 1 | 20,5 |
| 19 | 1 | 2 | 1 | 2 | 7,5 |
| 19 | 1 | 2 | 2 | 1 | 19,5 |
| 19 | 1 | 2 | 2 | 2 | 9,0 |
| 20 | 0 | . | . | . | 10,1 |
| 20 | 1 | 1 | 1 | 1 | 8,0 |
| 20 | 1 | 1 | 1 | 2 | 7,5 |
| 20 | 1 | 1 | 2 | 1 | 2,0 |
| 20 | 1 | 1 | 2 | 2 | 8,5 |
| 20 | 1 | 2 | 1 | 1 | 16,5 |
| 20 | 1 | 2 | 1 | 2 | 7,5 |
| 20 | 1 | 2 | 2 | 1 | 5,0 |
| 20 | 1 | 2 | 2 | 2 | 3,0 |
| 21 | 0 | . | . | . | 3,0 |
| 21 | 1 | 1 | 1 | 1 | 1,5 |
| 21 | 1 | 1 | 1 | 2 | 0,5 |
| 21 | 1 | 1 | 2 | 1 | 0,5 |
| 21 | 1 | 1 | 2 | 2 | 1,5 |
| 21 | 1 | 2 | 1 | 1 | 2,0 |
| 21 | 1 | 2 | 1 | 2 | 1,0 |
| 21 | 1 | 2 | 2 | 1 | 1,0 |
| 21 | 1 | 2 | 2 | 2 | 1,5 |
| 22 | 0 | . | . | . | 19,0 |
| 22 | 1 | 1 | 1 | 1 | 12,5 |
| 22 | 1 | 1 | 1 | 2 | 14,0 |
| 22 | 1 | 1 | 2 | 1 | 14,0 |
| 22 | 1 | 1 | 2 | 2 | 20,0 |
| 22 | 1 | 2 | 1 | 1 | 13,5 |
| 22 | 1 | 2 | 1 | 2 | 9,5 |
| 22 | 1 | 2 | 2 | 1 | 12,0 |
| 22 | 1 | 2 | 2 | 2 | 11,0 |
| 23 | 0 | . | . | . | 30,7 |
| 23 | 1 | 1 | 1 | 1 | 32,5 |
| 23 | 1 | 1 | 1 | 2 | 26,0 |
| 23 | 1 | 1 | 2 | 1 | 52,5 |
| 23 | 1 | 1 | 2 | 2 | 45,0 |
| 23 | 1 | 2 | 1 | 1 | 26,5 |
| 23 | 1 | 2 | 1 | 2 | 26,5 |
| 23 | 1 | 2 | 2 | 1 | 48,5 |
| 23 | 1 | 2 | 2 | 2 | 44,5 |
| 24 | 0 | . | . | . | 5,7 |
| 24 | 1 | 1 | 1 | 1 | 1,0 |
| 24 | 1 | 1 | 1 | 2 | 2,5 |
| 24 | 1 | 1 | 2 | 1 | 3,0 |
| 24 | 1 | 1 | 2 | 2 | 4,0 |
| 24 | 1 | 2 | 1 | 1 | 3,0 |
| 24 | 1 | 2 | 1 | 2 | 0,5 |
| 24 | 1 | 2 | 2 | 1 | 2,0 |
| 24 | 1 | 2 | 2 | 2 | 7,5 |
| 25 | 0 | . | . | . | 61,3 |
| 25 | 1 | 1 | 1 | 1 | 50,5 |
| 25 | 1 | 1 | 1 | 2 | 64,5 |
| 25 | 1 | 1 | 2 | 1 | 53,5 |
| 25 | 1 | 1 | 2 | 2 | 68,5 |
| 25 | 1 | 2 | 1 | 1 | 51,5 |
| 25 | 1 | 2 | 1 | 2 | 68,5 |
| 25 | 1 | 2 | 2 | 1 | 56,0 |
| 25 | 1 | 2 | 2 | 2 | 70,5 |
